# Different PSMA Radiopharmaceuticals: A Comparative Study of [^18^F]F-PSMA-1007, [^18^F]F-JK-PSMA-7, and [^99m^Tc]Tc-PSMA-I&S in the Skeletal System

**DOI:** 10.3390/ph17111458

**Published:** 2024-10-31

**Authors:** Zsófia Sára Mikó, László Varga, István Farkas, Gyula Tóth, Kristóf Apró, Barnabás Márk Révész, Gábor Sipka, Péter Gergő Tompa, Annamária Bakos, Tamás Czékus, Mátyás Bukva, László Pávics, Linda Varga, Anikó Maráz, Zsuzsanna Besenyi

**Affiliations:** 1 Department of Nuclear Medicine, University of Szeged, 6720 Szeged, Hungary; 2 Pozitron Diagnosztika Ltd., 1117 Budapest, Hungary; 3 Department of Immunology, University of Szeged, 6720 Szeged, Hungary; 4 Department of Oncotherapy, University of Szeged, 6720 Szeged, Hungary

**Keywords:** PSMA, radiopharmaceutical, SPECT, PET, UBU (unknown bone uptake), PSMA-1007, JK-PSMA-7, Tc-PSMA I&S

## Abstract

Background: Numerous PSMA-based tracers are used for diagnostic prostate cancer imaging, but comprehensive comparisons between multiple ligands are lacking. This study aimed to compare physiological skeletal uptake and tracer uptake in commonly recommended PSMA reference regions across three different PSMA ligands in prostate cancer patients. Methods: A total of 281 prostate cancer patients were included. Using PET and SPECT imaging, target volumes of interest were defined via a semiautomatic method, and standardized uptake values (SUV) were calculated for the skeletal system and reference regions (liver, spleen, parotid gland, and blood pool). Results: Significant differences in SUV uptake were observed, with [^18^F]F-PSMA-1007 showing higher SUV values in the skeletal system. The parotid gland displayed the highest variability in uptake, while the blood pool and liver exhibited more homogeneous uptake across patients. Conclusions: While radioligands behave similarly in bone regions, there are notable differences in SUV patterns, particularly for PSMA-1007, which showed higher bone uptake. Parotid gland uptake variability suggests a reconsideration of its suitability as a reference region, while the liver, spleen, and blood pool showed more consistent uptake. During comparison, the technetium-labeled SPECT ligand proved as similarly effective as the two PET ligands for diagnostic imaging.

## 1. Introduction

Prostate-specific membrane antigen (PSMA) is a transmembrane antigen and a key target for in vivo measurements using radiolabeled agents. Due to its minimal expression in healthy prostate tissues, PSMA serves as an ideal target molecule for imaging or therapeutic applications as a radiolabeled pharmaceutical, acting through an enzyme-substrate reaction. PSMA inhibitors specifically bind to PSMA, which is also referred to as glutamate carboxypeptidase II or *N*-acetylated α-linked acidic dipeptidase or *N*-acetyl-l-aspartyl-l-glutamate peptidase I. PSMA is a binuclear membrane-bound zinc protease and a metalloenzyme [1]. It has a molecular weight of 100–104 kDa, most of which is located in the extracellular space. Human PSMA is a protein composed of 750 amino acids and has a three-domain structure (Table 1) [2].

The PSMA enzyme is highly expressed on prostate tumor cells in most, but not all, patients. Its substrate, *N*-acetyl-l-aspartyl-l-glutamate (NAAG), binds to the extracellular part of the enzyme. During malignant transformation, PSMA moves from the prostate epithelium to the luminal surface, facilitating ligand interaction. While its exact function is unclear, the internalization of PSMA ligands via endocytosis suggests a transport role. PSMA expression is 100- to 1000-fold higher in prostate adenocarcinoma than in benign tissue and increases with tumor progression, particularly in metastatic castration-resistant prostate cancer (mCRPC) [3].

However, PSMA expression is not limited to prostate cancer. It is also overexpressed in the endothelial cells of other cancers and can be expressed in the endothelial cells of certain normal tissues, including the small intestine, renal nephrons, brain, and salivary glands [4].

Prostate-specific membrane antigen (PSMA)-based hybrid imaging is widely used for staging prostate cancer, though there is no consensus on the optimal tracer for assessing PSMA expression. In a theranostic approach, PSMA-directed imaging is essential for guiding radioligand therapy (RLT). PSMA PET/CT (or SPECT/CT) plays a critical role in assessing the presence and intensity of PSMA expression, which serves as a therapeutic target. A positive PSMA PET/CT scan is therefore required for inclusion in all [^177^Lu]Lu-PSMA-617 trials [5].

In clinical practice, improved logistics and availability have increased the use of ^18^F-labeled tracers for prostate cancer PET imaging. Despite this increase, ^99m^Tc remains widely used in diagnostic nuclear medicine due to its favorable properties. The number of clinical studies using ^99m^Tc exceeds that of PET agents, and [^99m^Tc]Tc-PSMA-I&S SPECT/CT may offer a cost-effective alternative to ^68^Ga or ^18^F-labeled PSMA PET/CT [6].

Recent studies have reported non-specific bone uptake on [^18^F]F-PSMA-1007 PET in a significant number of patients, although its clinical relevance remains unclear. This phenomenon may result in false-positive findings, potentially leading to inappropriate therapy [7,8,9]. However, despite the availability of relatively large observational data, non-specific bone uptake has not been systematically evaluated with other PSMA ligands, such as [^18^F]F-JK-PSMA-7 and [^99m^Tc]Tc-PSMA-I&S.

The aim of this retrospective analysis was to compare the physiological (non-pathological) skeletal tracer uptake of three different PSMA ligands in prostate cancer patients, using matched cohorts. Additionally, we aimed to assess the variability of tracer uptake in the commonly recommended PSMA reference regions (liver, spleen, parotid gland, and blood pool) with different radioligands.

## 2. Results

### 2.1. Patient Cohorts

In total, 281 patients were retrospectively included. The first study cohort involved 90 patients who underwent [^18^F]F-PSMA-1007 PET/CT, the second cohort consisted of 97 patients who received [^18^F]F-JK-PSMA-7 PET/CT, and the third cohort included 94 patients who received [^99m^Tc]Tc-PSMA-I&S SPECT/CT.

Patient characteristics are summarized in Table 2. All reported studies were conducted in accordance with the Helsinki Declaration and national regulations. The retrospective analyses were approved by the Ethics Committee of the University of Szeged (license no. 229/2017-SZTE).

### 2.2. Comparability of Patient Groups Examined with Different Radioligands

**Figure 1:**
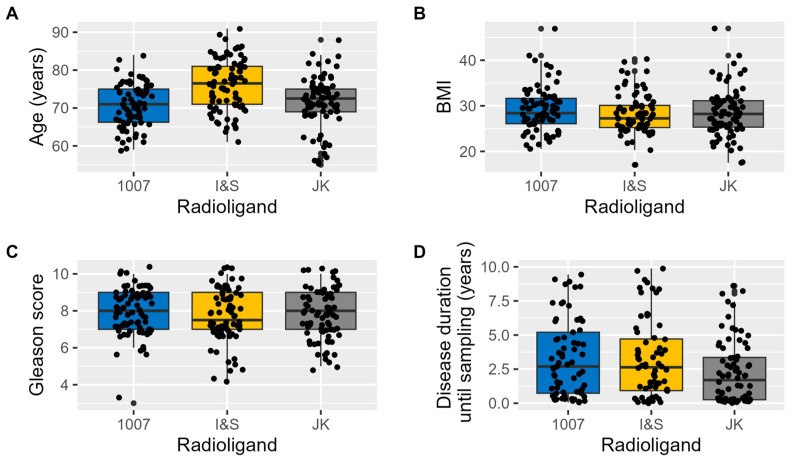
Basic clinical parameters of the radioligand patient groups. The boxplots illustrate the distribution of key clinical variables across patient groups examined with different radioligands. (**A**) shows the distribution of Age, (**B**) displays BMI, (**C**) depicts the Gleason score, and (**D**) represents Disease duration until imaging.

The groups examined with different diagnostic radioligands showed minimal differences across various basic clinical parameters (Figure 1). Furthermore, the multilevel correlation test revealed no significant correlation between these parameters and the SUV values obtained (Age: r = −0.04; BMI: r = −0.09; Gleason score: r = −0.03; Disease duration prior imaging: r = 0.03).

There were only minor differences between the groups in certain parameters that do not influence SUV values. Therefore, we consider the groups to be comparable.

### 2.3. Strong Correlation Among Maximum, Mean, and Peak SUV Values

**Figure 2:**
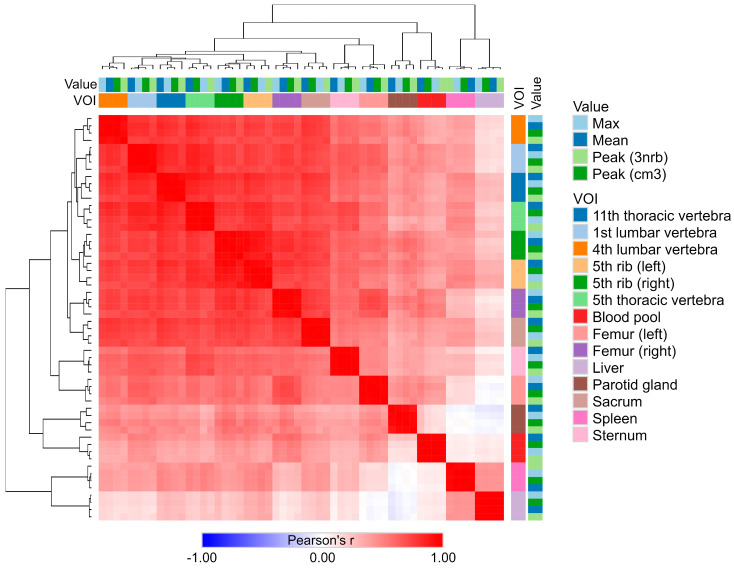
Heatmap and clustering analysis of SUV metrics across different VOIs. The heatmap illustrates the Pearson correlation coefficients (r) among different SUV metrics, including maximum, mean, and peak values, across different volumes of interest (VOIs). Each square in the heatmap represents the correlation between two variables, with red indicating positive correlations and blue indicating negative correlations. The color intensity corresponds to the strength of the correlation, with darker shades indicating stronger correlations. Hierarchical clustering on both axes organizes the VOIs and metrics, grouping together those with similar correlation patterns.

The results of the correlation analysis, combined with the heatmap and clustering (Figure 2), revealed that the individual SUV metrics (such as maximum, mean, and peak values) were highly correlated across all VOIs. The correlation coefficients (r) between these measures (intersections of columns and rows for a given VOI) ranged from 0.975 to 0.998, with all correlations being statistically significant (*p* < 0.001).

Notably, the SUV values observed in the liver VOI generally showed a negative correlation with several regions in the blood pool and the parotid gland.

Given the strong correlation between the SUV metrics, further analyses can be performed using only one selected measurement type. For the purposes of this study, subsequent analyses will focus on SUV peak (cm^3^) values.

### 2.4. Physiological Radiopharmacutical Uptake Homogeneity with Different Radiotracers in the Skeletal System and in Reference Regions

**Figure 3:**
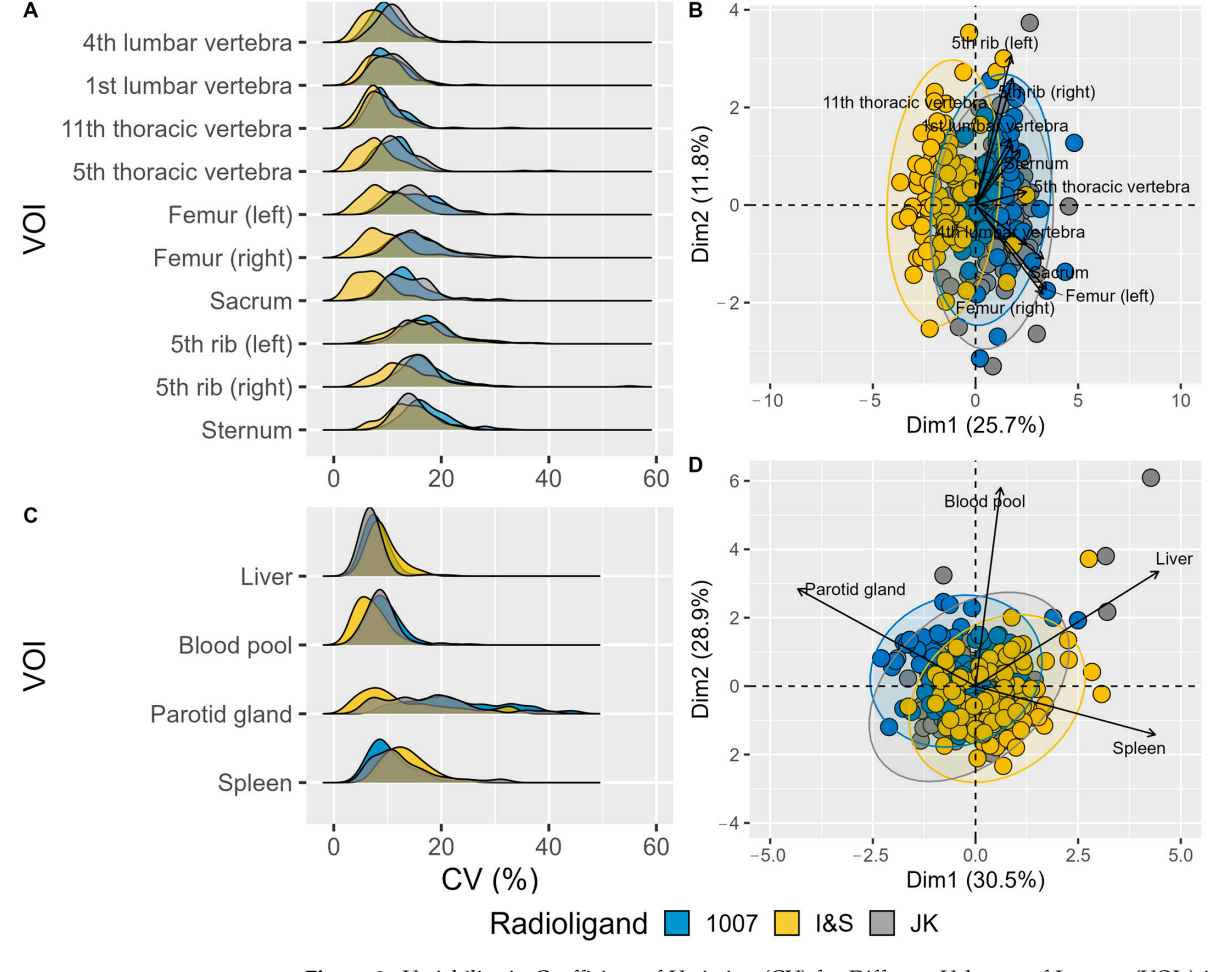
Variability in Coefficient of Variation (CV) for Different Volumes of Interest (VOIs) in Radioligand Uptake. (**A**) Density plots showing the distribution of CV (%) for various bone VOIs. Each VOI is represented separately along the *y*-axis, with the CV (%) plotted on the *x*-axis. (**B**) PCA biplot visualizing the relationships among skeletal VOIs based on their CV values. Different colors represent the three radioligands, and arrows indicate the direction and magnitude of the VOI contributions to the principal components. The length of each vector signifies the importance of the corresponding VOI in the PCA, with longer vectors indicating a greater contribution to the overall variance. Vectors pointing in similar directions suggest similar patterns of variation among VOIs. (**C**) Density plots showing the distribution of CV (%) for various reference VOIs. (**D**) PCA biplot visualizing the relationships among reference VOIs based on their CV values. (The more readable (**B**) panel can be found as Figure A1).

The radioligand uptake shows individual variability across different skeletal and reference VOIs, as depicted by the distribution of CV (%) values in Figure 3.

The lumbar vertebrae (1st and 4th) and thoracic vertebrae (5th and 11th) exhibit a range of CV values, indicating some level of variability among patients. The femur bones (left and right) display a similar distribution pattern, but with a slightly narrower range compared to the vertebrae. The ribs (left and right) and sternum also show distinct CV distributions, although the overall variability remains relatively comparable across these VOIs. Notably, the I&S radioligand demonstrates a shift toward lower CV values in several VOIs, while the 1007 and JK radioligands exhibit more similar distribution profiles, indicating comparable variability patterns between these two radioligands (Figure 3A).

Considering reference VOIs in the spleen, liver and blood pool, radioligand uptake is much more uniform across patients, whereas the parotid gland shows higher variability regardless of radioligand type (Figure 3C).

Overall, the I&S radioligand demonstrated the highest uptake homogeneity, whereas the 1007 and JK radioligands exhibited more similar variability profiles. The pattern revealed by Principal Component Analysis (PCA) and displayed in two dimensions (Figure 3B,D) was found to be statistically significant (*p* < 0.0001).

### 2.5. Distinct SUV Patterns Revealed by Different Radioligands Across Regions

The SUV values normalized to blood pool values reveal distinct distribution patterns of radioligands across various regions.

**Figure 4:**
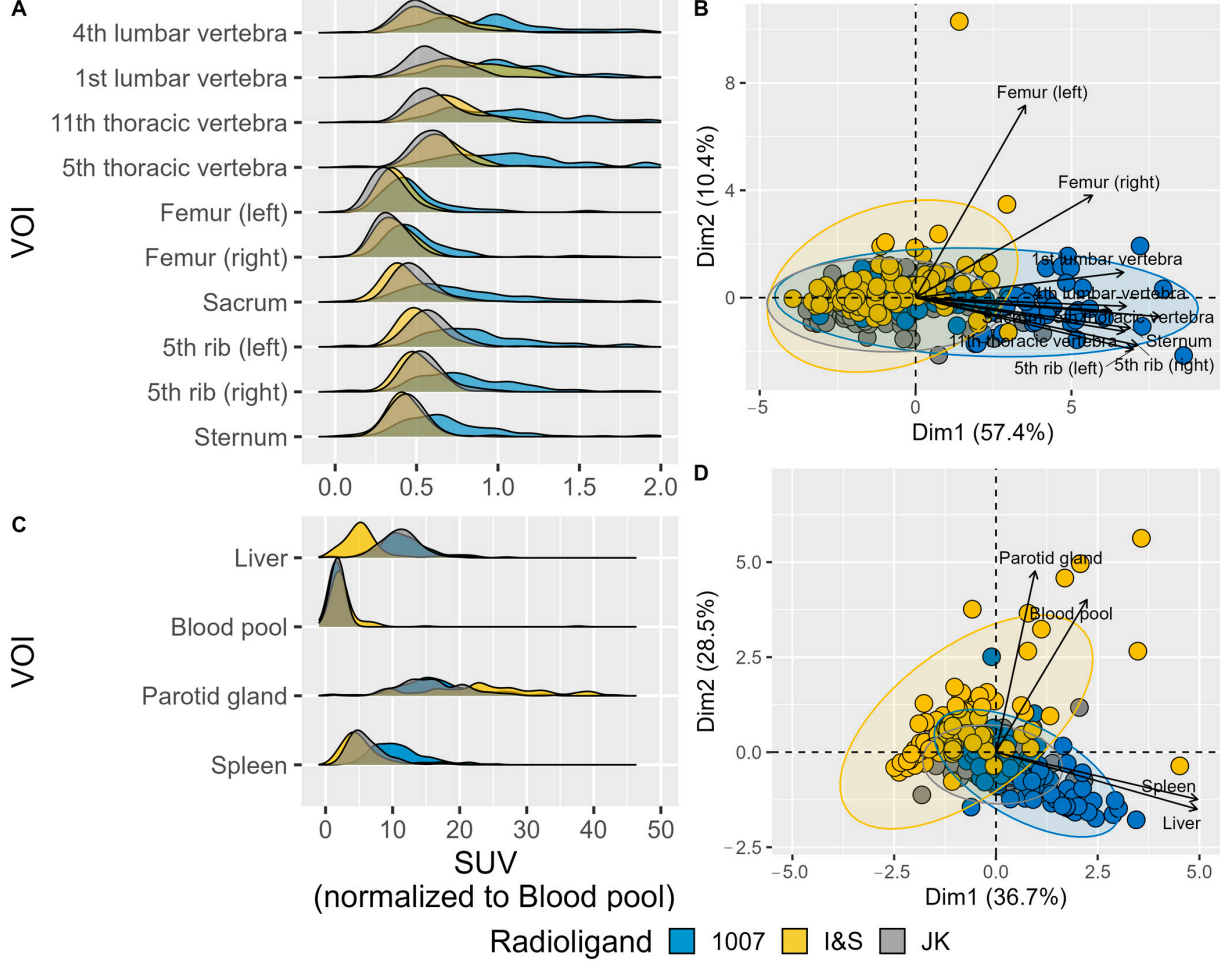
Normalized SUV peak values in different Volumes of Interest (VOIs). (**A**) Density plots showing the distribution of normalized SUV values for various bone VOIs. (**B**) PCA biplot visualizing the relationships among skeletal VOIs based on their peak SUV values. Different colors indicate the three radioligands, and arrows show the direction and magnitude of the VOI contributions to the principal components. The length of each vector represents the importance of the corresponding VOI in the PCA, with longer vectors indicating a greater contribution to the variance. The direction of the vectors indicates how each VOI is related to the principal components, with similar directions suggesting similar patterns of variation. (**C**) Density plots showing the distribution of peak SUV values (g/mL) for various reference VOIs. (**D**) PCA biplot visualizing the relationships among reference VOIs based on their peak SUV values. (The more readable (**B**) panel can be found as Figure A2).

In the bone regions (Figure 4A), the radioligands show similar behavior, with comparable peak SUV patterns across different bone regions. Despite the overall similarity, there are some differences among the radioligands. For instance, radioligand 1007 generally exhibits a higher SUV peak compared to I&S and JK. Meanwhile, JK shows a more consistent and narrower peak distribution across most bone regions, suggesting more uniform uptake. The I&S radioligand displays intermediate characteristics between 1007 and JK, with variability observed in certain regions such as the thoracic vertebrae.

For the blood pool, liver, spleen, and parotid gland regions (Figure 4D), the radioligands also show different distribution patterns. The spleen, blood pool and liver exhibit less variability in SUV values among the different radioligands, indicating a more homogeneous uptake between patients. The blood pool demonstrated a sharp peak for all radioligands, while the liver showed slightly broader peaks, with [^18^F]F-PSMA-1007 exhibiting the highest peak SUV values. On the other hand, the parotid gland shows higher variability in SUV values across radioligands, with 1007 having the highest peak SUV, followed by JK and I&S. This indicates that radioligand uptake in the parotid gland is more dispersed and less uniform compared to the blood pool, spleen, and liver across patients.

The PCA plots (Figure 4B,D) further highlight these differences. The PCA of the bone regions (Figure 4B) shows clustering that reflects the variability among the radioligands, with clear separation along the first principal component (Dim1), which accounts for 57.4% of the variance. This separation suggests significant differences in SUV uptake patterns among the radioligands. Similarly, the PCA of internal organs and glands (Figure 4D) revealed distinct clustering, with the first principal component (Dim1) explaining 36.7% of the variance.

Overall, the results suggest that, while radioligands exhibit similar behavior across different bone regions, significant differences in SUV uptake patterns were observed, with PSMA-1007 exhibiting higher peak SUV values. Among the blood pool, liver, spleen, and parotid gland regions, the blood pool and liver showed more consistent uptake across patients, while the parotid gland exhibited higher variability.

### 2.6. Correlation Between Bone Tracer Uptake and Radiation Therapy

According to the results of the generalized linear model, based on the available results, we cannot confirm that irradiation has an effect on the observed SUV (*p* = 0.301) and CV (*p* = 0.092) values in the irradiated lumbar vertebrae and sacrum. Similarly, the effect of ADT could not be confirmed on the basis of the available data (*p* = 0.311 for SUV and *p* = 0.223 for CV).

### 2.7. Limitations

Limitations of this study include its moderate sample size, its retrospective nature, and the lack of age-matched participants. However, all three patient groups had clinically comparable characteristics, including age. From a technical perspective, the different spatial resolutions of the PET and SPECT methods may be a limiting factor, although the functional resolution of the examinations is similar. Additionally, despite careful manual and semi-quantitative lesion and reference region definitions, the final contours could theoretically include pathological areas, such as micro-metastases.

## 3. Discussion

Our study provides a comprehensive comparison of three PSMA-based radiopharmaceuticals: [^18^F]F-PSMA-1007, [^18^F]F-JK-PSMA-7, and [^99m^Tc]Tc-PSMA-I&S, providing valuable insights into their uptake patterns and potential clinical applications.

In the skeletal system, [^18^F]F-PSMA-1007 consistently demonstrated higher SUV values compared to the other investigated tracers. The higher bone background uptake could lead to an increased rate of false-positive results, particularly in the case of benign bone lesions or degenerative changes. Several clinical studies have investigated the impact of UBU (unspecific bone uptake) with PSMA-1007 and concluded that the tracer remains clinically effective despite this phenomenon [7,8,9,10]. In a multicenter retrospective analysis, Grüning et al. investigated the commonly observed unspecific bone uptake in [^18^F]F-PSMA-1007 PET/CT scans of 348 prostate cancer patients and found that UBU was present in 51% of patients. The incidence of UBU did not correlate with PSA levels, Gleason score, tumor stage, or patient age. Interestingly, UBU was significantly more common on digital PET/CT scans compared to analog PET/CT. [^18^F]F-PSMA-1007 PET/CT scans, especially with digital scanners, often show non-specific bone lesions that should be interpreted cautiously to avoid over-staging patients. Despite the many advantages of [^18^F]F-PSMA-1007, this radiotracer has certain limitations [8]. We hypothesize that the higher incidence of unspecific bone uptake observed with the PSMA-1007 tracer, as supported by our study, may be attributed to its inherently higher bone uptake compared to other PSMA ligands. This increased uptake likely contributes to the increased incidence of UBU. Another study by Arnfield et al. concluded that a higher rate of UBU holds no clinical significance and does not significantly affect the diagnostic efficacy of the tracer [9].

Another potential cause of the higher incidence of UBU could be osteoporosis. In a retrospective study, Ninatti et al. explored the potential association between osteoporosis and unspecific [^18^F]F-PSMA-1007 bone uptake in prostate cancer patients. They analyzed 77 treatment-naïve patients who underwent [^18^F]F-PSMA-1007 PET for staging and it was observed in 38% of patients. The authors investigated markers (such as blood count) that could correlate with bone mineral density. Although not statistically significant, patients with unspecific bone uptake also had lower BMI and bone density values. The authors concluded that non-specific bone uptake of [^18^F]F-PSMA-1007 may be associated with osteoporosis, possibly due to the complex interplay between the immune system and bone remodeling cells. This exploratory analysis suggests a possible link between osteoporosis and unspecific [^18^F]F-PSMA-1007 bone uptake in prostate cancer patients [10]. Direct bone densitometry was not performed in our patient population, which is a limitation that future prospective studies could address. The impact of androgen deprivation therapy (ADT) on bone health is well established, with long-term treatment known to increase bone porosity. However, in our patient group, no significant association was observed between the use of hormone therapy and bone radiopharmaceutical uptake.

An increasing number of studies have demonstrated the efficacy of ^99m^Tc-labeled SPECT tracers in prostate cancer imaging [6,11,12,13,14,15]. A notable finding of our investigation was that [^99m^Tc]Tc-PSMA-I&S, despite being a SPECT tracer, showed comparable efficacy to the ^18^F-labeled PET ligands in terms of uptake patterns and homogeneity. This suggests that SPECT-based PSMA imaging could potentially offer a cost-effective alternative to PET in certain clinical scenarios, without significantly compromising diagnostic performance. This has important implications for expanding access to PSMA-based imaging, especially in resource-limited settings. In addition, technological advances and improved imaging resolution in SPECT have largely minimized the differences compared to PET, enabling comparable results between SPECT and PET imaging using different radiotracers. However, some limitations remain due to differences in tracer characteristics, particularly with respect to patient comfort. For instance, Tc labeled radiopharmaceuticals require a longer uptake time, (minimum 5 h),whereas PET tracers can be imaged as early as 90–120 min post-injection. The advantage of longer uptake time for Tc–labeled tracers is their applicability in intraoperative radio-guided surgery [16].

Efforts to standardize reporting and interpretation of PSMA hybrid imaging are actively underway, as standardized approaches are increasingly adopted in diagnostic procedures. In PSMA ligand PET/CT, several frameworks for standardized reporting and therapy response evaluation have been proposed, and these frameworks are expected to evolve further as clinical experience increases. Given the differences in biodistribution between different PSMA ligands, multiple reference regions—including the liver, spleen, and blood pool—are frequently used to assess relative radiopharmaceutical uptake [17].

Additionally, some guidelines have suggested using the salivary glands as a reference region, although this approach is still under evaluation [18]. Our findings highlight the need for careful, tracer-specific consideration when interpreting results and selecting reference standards due to observed differences in uptake patterns across reference regions. The variability of parotid gland uptake for all three tracers challenges its suitability as a reference region. In contrast, a recent retrospective multicenter study aimed to establish a PSMA PET tumor-to-salivary gland ratio to predict outcomes following [^177^Lu]PSMA therapy [19]. However, our findings indicate that the parotid gland should be carefully reconsidered as a reference region, as it showed the greatest individual variability among all tracers in the prostate cancer patient group. In contrast, the blood pool and liver exhibited more homogeneous uptake patterns, confirming their suitability as reference regions for comparing standardized uptake values (SUV) and for inter-patient comparisons.

Future research on different PSMA tracers should include prospective studies to validate these findings, as well as studies directly comparing the diagnostic accuracy of these tracers in detecting prostate cancer lesions, particularly in cases of biochemical recurrence or small-volume metastatic disease.

## 4. Materials and Methods

### 4.1. Patient Population

Patients who underwent [^18^F]F-PSMA-1007, [^18^F]F-JK-PSMA-7 PET/CT, or [^99m^Tc]Tc-PSMA-I&S were retrospectively considered for this analysis. Inclusion criteria were of men with histologically proven prostate cancer. Exclusion criteria included patients with disseminated high-volume bone metastases (affecting almost all vertebrae, ribs, pelvis, etc.) on PSMA scans, the presence of bone implants, or known skeletal fractures.

Indications for PSMA imaging were:

(i) primary staging of histologically confirmed high- or intermediate-risk prostate carcinoma prior to radiation therapy or surgery (i.e., PSA (Prostate-Specific Antigen) > 20 ng/mL, Gleason score ≥ 4 + 3 = 7, or clinical stage ≥ T3), or
(ii) restaging related to biochemical recurrence (PSA ≥ 0.2 ng/mL after prostatectomy or ≥2.0 ng/mL above the nadir after radiotherapy).

Additional recorded variables included Gleason score, ISUP (International Society of Urological Pathology) grade at diagnosis, prior radiotherapy and androgen deprivation therapy (ADT), and the most recent PSA value.

### 4.2. Acquisition

#### 4.2.1. PSMA SPECT/CT

The radiosynthesis of PSMA-I&S has been previously reported by Robu et al. [6]. The [^99m^Tc]Tc-PSMA-I&S kit was labeled with 5.7 ± 0.88 GBq of [^99m^Tc]pertechnetate. Radiochemical purity assays were performed using instant thin-layer chromatography on silica gel, with butan-2-one as the mobile phase. The mean radiochemical purity was 99.6 ± 0.4%. The mean activity of the labeled compound was 668 ± 95 MBq for ^99m^Tc-mas3-y-nal-k (Sub-KuE).

**Figure 5:**
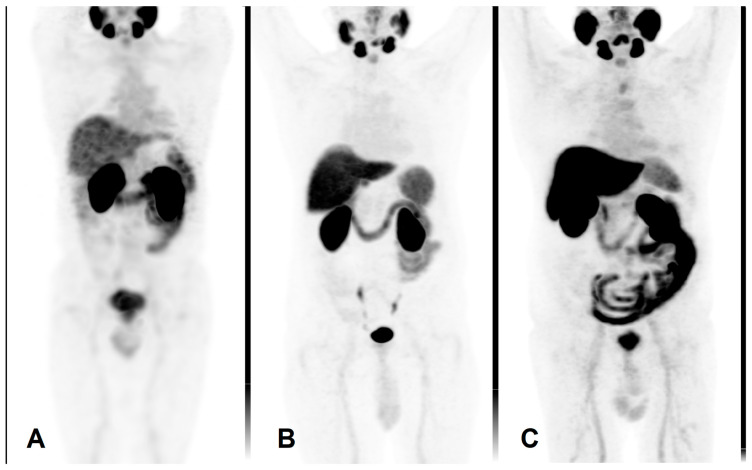
Maximum intensity projection PET images using different radiopharmaceuticals. (**A**) [^99m^Tc]Tc-PSMA-I&S, (**B**) [^18^F]PSMA-1007, (**C**) [^18^F]F-JK-PSMA-7.

Mean activity of 666 ± 102 MBq [^99m^Tc]Tc-PSMA-I&S (Institute of Isotopes Co., Ltd., Budapest, Hungary) was administered intravenously. Prior to imaging, patients were given oral contrast material (1000 mL of polyethylene glycol solution) to drink continuously, starting 60 min before the examination. Scans were performed on an integrated whole-body SPECT/CT system (Mediso AnyscanTRIO, Mediso Medical Imaging Systems Ltd., Budapest, Hungary). Whole-body SPECT imaging was carried out 6 h after radiopharmaceutical administration, with acquisition parameters set at 360° rotation, 96 projections, 10 s per frame, matrix size 128 × 128, and reconstructed pixel size of 4.22 mm. SPECT data acquisition was supplemented by a low-dose CT scan (120 kV and 70 mA·s). An illustrative image is presented in Figure 5A.

#### 4.2.2. PSMA PET/CT

The synthesis of [^18^F]F-JK-PSMA-7 was achieved by a one-step radio-fluorination reaction, as described by Arndt et al. [20].

The radiosynthesis of [^18^F]F-PSMA-1007, which resembles the structure of the therapeutic agent PSMA-617 [21], was performed using a simplified one-step automated method, which was followed by the direct radio-fluorination of the precursor (5-((*S*)-4-carboxy-1-((*S*)-4-carboxy-1-(4-((*S*)-1-((*S*)-5-carboxy-5-(3-((*S*)-1,3-dicarboxypropyl)-ureido)pentylamino)-3-(naphthalen-2-yl)-1-oxopropan-2-ylcarbamoyl)benzylamino)-1-oxobutan-2-ylamino)-1-oxobutan-2-ylcarbamoyl)-*N*,*N*,*N*-trimethylpyridin-2-aminium acetate) [22]. The precursor and the cold reference standard of PSMA-1007 was purchased from ABX GmbH, Radeberg, Germany, while the ^18^F fluoride was produced via the 18O(p,n)^18^F nuclear reaction through proton irradiation of enriched (98%) ^18^O water using an Eclipse HP cyclotron. The radiosynthesis was carried out by utilizing a cassette based AllInOne synthesizer (Trasis S.A., Ans, Belgium), with a typical production yield of 40 ± 5% (non-decay corrected). The final product underwent standard quality control procedures. The [^18^F]F-PSMA-1007 was analyzed using radio-HPLC and it was compared to a reference sample of PSMA-1007, confirming a radiochemical purity of ≥97%.

The same imaging protocol was used with both ^18^F-labeled radiopharmaceuticals, [^18^F]F-JK-PSMA-7 and [^18^F]F-PSMA-1007.

The PET/CT scans were performed using a GE Discovery IQ Gen 2 PET/CT System (GE Healthcare, Chicago, IL, USA). Acquisitions began 90 min post-injection of 3.7 MBq/kg of the intravenous radiopharmaceutical. Prior to imaging, patients were administered a positive iodinated oral contrast agent, which they were instructed to drink continuously starting 60 min before the scan. The PET scan was performed in three-dimensional (3D) mode, with each bed position scanned for 2.5 min. The field of view was 20 cm with a 30% overlap between bed positions. Data acquisition was complemented by a low-dose plain CT scan (120 kV and 70 mAs) for mapping purposes. The reconstructed pixel size was 3.65 mm. Routine whole-body mapping was performed, covering the area from the base of the skull to the upper third of the thigh. These are illustrated in Figure 5B,C.

### 4.3. Imaging Analysis

**Figure 6:**
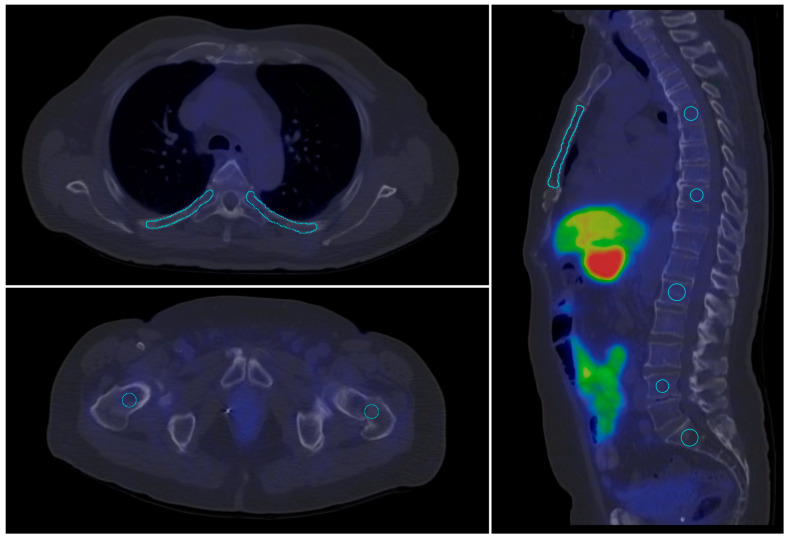
Contoured skeletal volumes of interest (VOI) (blue lines) in standard locations on a [^18^F]F-JK-PSMA PET/CT scan.

For the quantitative evaluation of tracer uptake on PET and SPECT slices, target volumes of interest (VOIs) were defined using a semi-automated method, excluding any visible abnormalities on the corresponding CT slices. The VOI segmentation were performed in InterView^TM^ FUSION software (Mediso Medical Imaging Systems Ltd., Budapest, Hungary).To assess healthy bone uptake, 10 different bone contours were outlined at predefined, standard locations. Healthy parotid gland, aortic arch (blood pool), liver and spleen tissues were selected as reference regions. VOIs were defined on both SPECT and PET scans in areas without visible abnormalities on CT slices (see Table 3). A visual representation of this process is shown in Figure 6.

The SUV_max_, SUV_mean_, SUV_SD_, SUV_peakcm3_ and SUV_peak3nbr_ were determined by defining a volume of interest (VOI) around the target lesions.

### 4.4. Data Analysis

The first step in the analysis was to compare the groups examined with different radioactive substances based on clinical parameters (age, BMI (body mass Index), Gleason score, and disease duration prior imaging) to ensure their comparability. This process included graphical representation of the parameters and multilevel correlation analysis to explore their relationship with SUV values.

Next, we assessed the homogeneity of radioligand uptake in each VOI by calculating the Coefficient of Variation (CV), using the mean and standard deviation of SUV values. This approach helps to separate the extent of variation from the scale of the original values. A higher CV indicates greater relative inhomogeneity, whereas a lower CV suggests more consistent uptake of the radioligand within the given VOI.

Following the CV analysis, Principal Component Analysis (PCA) was performed to visualize global patterns in radioligand uptake. The PCA results were presented as a biplot to illustrate these patterns. Prior to PCA, the dataset was scaled to standardize the variables.

We then examined the relationship between different SUV metrics, such as SUV_max_, SUV_mean_, SUV_SD_, SUV_peakcm3_ (the maximum average SUV within a 1 cm^3^ sphere centered in the highest uptake region of the VOI), and SUV_peak3nbr_ (the maximum average of the peak and the 3 highest neighboring SUVs within the VOI). The aim of this analysis was to determine the extent to which these different metrics are comparable and interchangeable in clinical practice. The SUV metrics in the data matrix were correlated across the patient cohort using the Pearson correlation method. The resulting correlation matrix was then clustered using Euclidean distances and complete linkage, and the findings were visualized in heatmaps.

Additionally, the effects of radiotherapy on SUV and CV values (in the lumbar vertebrae and sacrum) were evaluated using a Generalized Linear Model (Gamma family, identity link).

The analysis was performed using RStudio 2023.06.1 software, utilizing the ggplot2 (3.5.1), correlation (0.8.5), FactoMineR (2.11), factoextra (1.0.7), and ggridges (0.5.6) packages.

## 5. Conclusions

Our results demonstrated that, while radioligands exhibit similar behavior across different bone regions, notable differences exist in their SUV uptake patterns. Specifically, PSMA-1007 showed higher peak SUV values compared to other radioligands investigated. Interestingly, the parotid gland exhibited the highest individual variability across all tracers, suggesting that the use of the salivary gland as a reference region may require careful reconsideration. Our results indicate that, while there are observable differences in tracer behavior concerning bone uptake, these differences are not clinically significant.

The technetium-labeled SPECT ligand showed comparable efficacy to the two PET ligands for diagnostic imaging. Its uptake and homogeneity closely resembled the uptake patterns observed with the PET ligands, further confirming its suitability for clinical diagnostics.

## Figures and Tables

**Table 1:** Location and composition of PSMA [2].

	Location	Composition
Portion 1	Extracellular	707 amino acids
Portion 2	Transmembrane	24 amino acids
Portion 3	Intracellular	19 amino acids

**Table 2:** Patient characteristics summary.

Variables		Overall	JK-PSMA-7	PSMA-1007	PSMA-I&S
Number of patients		281	97	90	94
Age (median, IQR) (years)		73 (9)	73 (6)	71 (10)	76 (11)
BMI (median, IQR) (kg/m^2^)		28.0 (5.5)	28.1 (5.7)	28.1 (5.2)	27.8 (5.1)
PSA (median, IQR) (ng/mL)		2.9 (12.9)	3.7 (13.0)	1.7 (9.5)	4.2 (17.4)
Gleason-score (*n*; %)	≤6	36 (13)	18 (19)	7 (8)	11 (12)
	7	84 (30)	22 (23)	28 (31)	34 (36)
	8–10	140 (50)	48 (49)	48 (53)	44 (47)
ISUP grade (*n*, %)	I	35 (12)	18 (19)	7 (8)	10 (11)
	II	47 (17)	13 (13)	10 (11)	24 (26)
	III	32 (11)	8 (8)	17 (19)	7 (7)
	IV	50 (18)	19 (20)	18 (20)	13 (14)
	V	89 (32)	29 (30)	30 (33)	30 (32)
iPSA (median, IQR) (ng/mL)		18.4 (41.0)	16.6 (45.6)	18.6 (33.3)	20.4 (42.1)
ADT+ (*n*; %)		185 (66)	53 (55)	42 (47)	90 (96)
Radiation therapy+ (*n*; %)		92 (33)	33 (34)	37 (41)	22 (23)

Abbreviations: IQR—Interquartile Range, BMI—Body Mass Index, PSA—Prostate-Specific Antigen, ISUP—International Society of Urological Pathology, iPSA—Initial Prostate-Specific Antigen, ADT—Androgen Deprivation Therapy.

**Table 3:** Reference regions and bone VOI (volume of interest) definition.

Region 1	VOI
parotid gland	iso-count 3D VOI
blood pool	sphere diameter = 15 mm
liver	sphere diameter = 30 mm
right and left femoral bone	sphere diameter = 15 mm
spleen	sphere diameter = 30 mm
sacrum	sphere diameter = 15 mm
lumbar vertebra 1, 4	sphere diameter = 15 mm
thoracal vertebra 5, 9	sphere diameter = 15 mm
left and right rib 5, 9	freehand brush tool radius ~ 3 mm
sternum corpus	on sagittal plane radius ~ 3 mm

**Figure A1:**
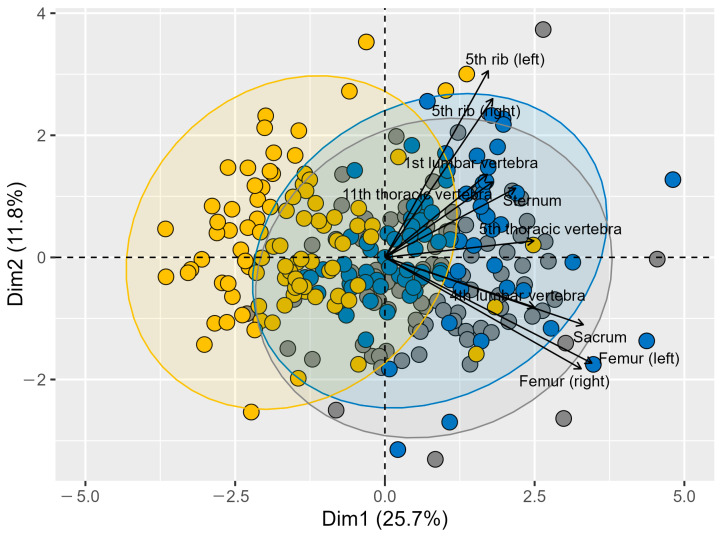
PCA biplot visualizing the relationships among skeletal VOIs based on their CV values. Different colors represent the three radioligands, and arrows indicate the direction and magnitude of the VOI contributions to the principal components. The length of each vector signifies the importance of the corresponding VOI in the PCA, with longer vectors indicating a greater contribution to the overall variance. Vectors pointing in similar directions suggest similar patterns of variation among VOIs.

**Figure A2:**
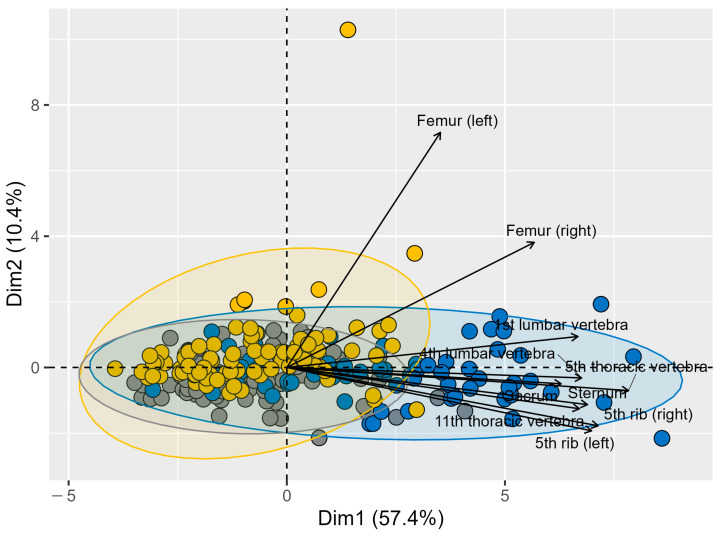
PCA biplot visualizing the relationships among skeletal VOIs based on their peak SUV values. Different colors indicate the three radioligands, and arrows show the direction and magnitude of the VOI contributions to the principal components. The length of each vector represents the importance of the corresponding VOI in the PCA, with longer vectors indicating a greater contribution to the variance. The direction of the vectors indicates how each VOI is related to the principal components, with similar directions suggesting similar patterns of variation.

## Data Availability

Data are available from the authors upon request.
